# Informing the management of acute malnutrition in infants aged under 6 months (MAMI): risk factor analysis using nationally-representative demographic & health survey secondary data

**DOI:** 10.7717/peerj.5848

**Published:** 2019-04-15

**Authors:** Marko Kerac, Severine Frison, Nichola Connell, Bethan Page, Marie McGrath

**Affiliations:** 1 Department of Population Health, London School of Hygiene & Tropical Medicine, London, UK; 2 MARCH Centre, London School of Hygiene & Tropical Medicine, London, UK; 3 Department of Global Health, Save the Children USA, Washington, D.C., USA; 4 Independent, London, UK; 5 Emergency Nutrition Network, Oxford, UK

**Keywords:** Malnutrition, Severe malnutrition, Wasting, Severe acute malnutrition, Infant, Risk factor, Demographic and health survey, Breastfeeding

## Abstract

**Background:**

Tackling malnutrition is a global health priority, helping children both survive and thrive. Acute malnutrition (wasting) in infants aged under 6 months (u6m) is often neglected. Worldwide, some 8.5 million infants u6m are affected yet recent World Health Organization malnutrition guidelines highlight numerous evidence gaps on how to best manage them. To inform future research, policy and programming, we aimed to identify risk factors associated with infant u6m wasting.

**Methods:**

We did secondary data analysis of nationally representative, cross sectional Demographic and Health Surveys conducted in the last 10 years. We compared wasted infants u6m (weight-for-length <−2 *z*-scores) vs. non-wasted (weight-for-length ≥−2 *z*-score). We used simple and adjusted (for infant age, sex, socio-economic status) logistic regression to calculate odds of wasting associated with risk factors spanning three broad categories: household-related; maternal-related; infant-related.

**Results:**

We analysed 16,123 infants u6m from 20 countries. Multiple risk factors were statistically associated with wasting. These included: poverty (Odds ratio, OR 1.22 (95% CI [1.01–1.48], *p* = 0.04)); low maternal body mass index (adjusted OR 1.53(1.29–1.80, *p* < 0.001); small infant size at birth (aOR 1.32(1.10–1.58, *p* < 0.01)); delayed start of breastfeeding (aOR 1.31(1.13–1.51, *p* < 0.001)); prelacteal feed (aOR 1.34(1.18–1.53, *p* < 0.001)); recent history of diarrhoea (aOR 1.37(1.12–1.67, *p* < 0.01)); mother disempowered (experiences violence; does not make decisions about health issues; does not engage with health services such as antenatal care, does not give birth in a health facility). ‘Protective’ factors associated with significantly decreased odds of infant u6m wasting included: educated mother (OR 0.64(0.54–0.76, *p* < 0.001)); mother in work (OR 0.82(0.72–0.94, *p* < 0.01)); currently breastfed (aOR 0.62(0.42–0.91, *p* = 0.02)), exclusively breastfed (aOR 0.84(0.73–0.97, *p* = 0.02).

**Discussion:**

Infant u6m wasting is a complex, multifactorial problem associated with many risk factors; knowing them will help shape international and national management strategies. Whilst our observational study cannot prove causation, many factors identified are biologically plausible and/or socially important. They should be considered when assessing and managing infants u6m. Although supporting breastfeeding is core to future interventions, this alone is unlikely to be sufficient; strategies should involve multiple sectors, beyond just health and nutrition. By noting our results, future intervention studies could focus resources and maximise chances of achieving impact.

## Background

Tackling malnutrition plays a major role in child survival (Black et al., 2013) and also, as highlighted in Global Strategy for Women’s, Children’s and Adolescents’ Health (2016), it also has a core role in the emerging ‘Thrive’ agenda. Short-term effects such as increased risk of infectious disease have long been recognised (Tomkins, 2000). There is also now increasing evidence of long-term consequences, including for the growing epidemic of non-communicable disease (Bhutta, 2013; Victora et al., 2008; Gluckman, Hanson & Pinal, 2005; Hawkes & Popkin, 2015).

Wasting, the main manifestation of acute malnutrition, affects over 52 million children worldwide (Black et al., 2013) and is recognised in target 2.2 of the Sustainable Development Goals: ‘*by 2030, end all forms of malnutrition, including achieving by 2025 the internationally agreed targets on stunting and wasting [acute malnutrition] in children under five years of age*’, (United Nations, 2015), (No Wasted Lives, 2018). Latest December 2013 World Health Organization (WHO) guidelines on the Management of Severe Acute Malnutrition (SAM) are a key step towards tackling wasting because they outline the latest, evidence-based approaches to the care of affected infants and children (WHO, 2013). For the first time at international level, they also include a chapter dedicated to infants aged under 6 months (henceforth infants u6m) (WHO, 2013). Almost 4 million infants u6m worldwide are severely wasted and 5 million are moderately wasted (Kerac et al., 2011). Yet, despite these numbers and despite their being the most vulnerable and at-risk of death (Grijalva-Eternod et al., 2016), the quality of evidence underpinning their management is ‘very low’ (WHO, 2013). Recently, the ‘No Wasted Lives’ global consortium of agencies dedicated to tackling severe malnutrition has flagged the management of infants u6m as one of just six priority areas urgently needing further evidence (No Wasted Lives, 2018).

In 2015, an international Child Health and Nutrition Research Initiative exercise consulted a wide range of international experts and practitioners on research priorities for improving the management of acutely malnourished infants u6m (Angood et al., 2015). A long list of possible research questions was ranked based on (i) answerability; and likelihood of the future intervention arising from the research being: (ii) efficacious; (iii) effective; (iv) deliverable; (v) sustainable; (vi) reducing disease burden. The top-ranked question identified in that consultation was ‘How should infant SAM be defined?’ Second was ‘What are the key opportunities/timings where infant SAM management can be incorporated with other healthcare programmes?’ Third was ‘What are the priority components of a package of care for outpatient treatment of infant u6m SAM?’ (Angood et al., 2015). For all these priority questions—and for many others on the list—better understanding of the risk factors underlying infant u6m malnutrition is vital. However, data focusing on this is scarce; to improve future outcomes for this vulnerable group, better evidence is urgently needed (Kerac et al., 2015).

In this paper, our aim was to use existing large national datasets to identify which of a wide variety of biologically plausible risk factors are actually associated with acute malnutrition in infants u6m. These data are needed to inform assessment and management approaches in the immediate/short term and shape intervention studies in the medium term. The long term goal is to ensure effective, evidence based treatment of acutely malnourished and at-risk infants u6m.

## Methods

### Study design and country inclusion criteria

We conducted a secondary data analysis of Demographic and Health Survey (DHS) datasets. DHS are large, nationally representative household surveys regularly conducted in over 90 developing countries. They follow a common, standardised methodology (two stage cluster sampling) and are extensively used in global health and nutrition to support ‘policy formation, program planning and monitoring and evaluation’ (DHS, 2017d). Questionnaires including a household questionnaire and a women’s questionnaire (containing child health topics) are administered—following informed consent—by local field staff in each country, in local languages, using ‘standard procedures, methodologies, manuals and videos to guide the survey process’ (DHS, 2017c). Full survey questionnaires for each country are available in the annexes of each final country report.

Following the approach from a previous paper exploring the prevalence of acute malnutrition in infants u6m (Kerac et al., 2011), our sampling frame was the 36 countries described by the Lancet Nutrition series as having the highest burden of malnutrition (Black et al., 2008). From this, we selected countries with: (a) an available survey done in the last 10 years (DHS phase 5 or 6); (b) available anthropometric data on infants u6m and children, including sex, age, weight and length. Where more than one survey had been done in the last 10 years, we included only the most recent, hence had one survey only per country.

### Participants

Only infants u6m were included in our analysis. We compared those with acute wasting (weight-for-length *z*-score, WLZ <−2) with those who were not wasted (WLZ ≥−2). We used WHO growth standards for our analysis (WHO, 2017). Wasting was further subdivided into severe (WLZ <−3) and moderate (WLZ ≥−3 to <−2). Moderate wasting corresponds to moderate acute malnutrition (MAM) whilst severe wasting approximates SAM (WHO & UNICEF, 2009); DHS do not collect data on oedema so severe wasting was the closest measure of SAM (SAM includes oedematous malnutrition as well as severe wasting) (Frison, Checchi & Kerac, 2015).

### Variables and data handling

Demographic and Health Survey country datasets are formatted in a consistent structure using common variable headings, with questions asked and coded in the same way (translation into local language allowing) in each country (DHS, 2017b). Variables included age, sex, weight and/length; these four variables were used to calculate anthropometric indices, our main outcome variables.

Potential explanatory variables were grouped under: household characteristics; maternal characteristics; infant characteristics. Our rationale for these three categories was that they all plausibly affect infant nutrition and might be amenable to change in future intervention programmes. Since this was a hypothesis-generating exploratory study and we did not know exactly which aspects of household environment, which maternal characteristics and which infant characteristics are most closely associated with infant malnutrition, we deliberately chose all available variables and excluded none.

Detailed variable definitions are given on DHS website and the exact phrasing of questions can be seen in country questionnaires (DHS, 2017c). Among the key variables we used in our adjusted analysis was wealth index: this is expressed in quintiles, is already calculated in DHS datasets, and is a ‘composite measure of a household’s cumulative living standard….calculated using data on….selected assets, such as televisions and bicycles; materials used for housing construction; and types of water access and sanitation facilities’. Full details of wealth index for each country is described on the DHS website (DHS, 2017e). Also important to note is that some variables are as-reported rather than objectively measured/verified and therefore some are more vulnerable to bias than others. For instance, ‘size at birth’ is a perceived/reported by the mother because many countries do not have birth weight widely recorded—research suggests that this underestimates true low birth weight (Blanc & Wardlaw, 2005).

Using Stata version 13.1 (StataCorp. 2013. Stata Statistical Software: Release 13. College Station, TX: StataCorp LP) we merged individual country files into one large dataset. This was then cleaned by excluding records with missing or extreme values. Infants u6m were excluded if any of the following were missing: age; sex; weight; length. Those with biologically unlikely extreme values (‘flags’) were also excluded from analysis following standard WHO ‘cleaning rules’ (Crowe et al., 2014): weight-for-age *z*-score (WAZ)<−6.0 or WAZ>+5.0, length-for-age *z*-score (LAZ)<−6.0 or LAZ>+6.0, WLZ<−5.0 or WLZ>+5.0 SD. These more likely represent measurement or data processing errors rather than a truly very large or small infant.

*Z*-scores were generated from raw age, weight and length variables using the WHO’s ‘Child Growth Standards’ macro (WHO, 2011).

### Study size

This was constrained by available DHS datasets and by the number of infants u6m in each. Surveys vary by country, with larger countries generally having larger DHS sample sizes. All are designed to be more than just nationally representative of key indicators, rather to also ‘compare the survey results for different characteristics such as urban and rural residence, different administrative or geographic regions, or different educational levels of respondent’ (ICF International, 2012).

### Data analysis

We used Stata’s survey (svy) commands for our main analysis to account for the DHS survey design.

Firstly, we computed the prevalence and 95% confidence intervals of severe, moderate and overall wasting based on WLZ for each country and for all countries combined. For the overall figure and for logistic regressions (described below) we accounted for different population size using population estimates from the World Bank (www.populationpyramid.net, United Nations, Department of Economic and Social Affairs, Population Division. World Population Prospects).

Our main analysis consisted of multiple binary logistic regression. Overall wasting is presented in the main results tables as the binary dependent variable: this is subdivided into severe wasting and moderate wasting in Supplementary Tables. Numerous independent variables (risk factors) were explored, under three general categories: household characteristics; maternal characteristics; infant characteristics.

We also looked at risk factors adjusted for infant age group, sex and socio-economic status (this included wealth index, maternal education, mother working or not; mother in union or not). All these may plausibly contribute to infant wasting, hence are important confounders and need to be taken into account when assessing other variables.

Odds ratios and 95% confidence intervals were calculated and are presented. Statistical significance was taken as *p* < 0.05. Stata (StataCorp. 2013. Stata Statistical Software: Release 13. College Station, TX: StataCorp LP) was used for all analyses.

### Ethics, consent and permissions

Demographic and Health Survey are a well-established and well-respected global initiative conducted with all appropriate in-country permissions and informed consents. For inclusion of infants u6m, their main carers (usually mothers) would have given informed, usually written, consent. We registered our project on the DHS website (DHS, 2017a) and were granted permission from DHS to download the datasets in Stata format. Our project was also reviewed and approved by the London School of Hygiene and Tropical Medicine Observational Research Ethics Committee, reference number 10401.

## Results

**Figure 1:**
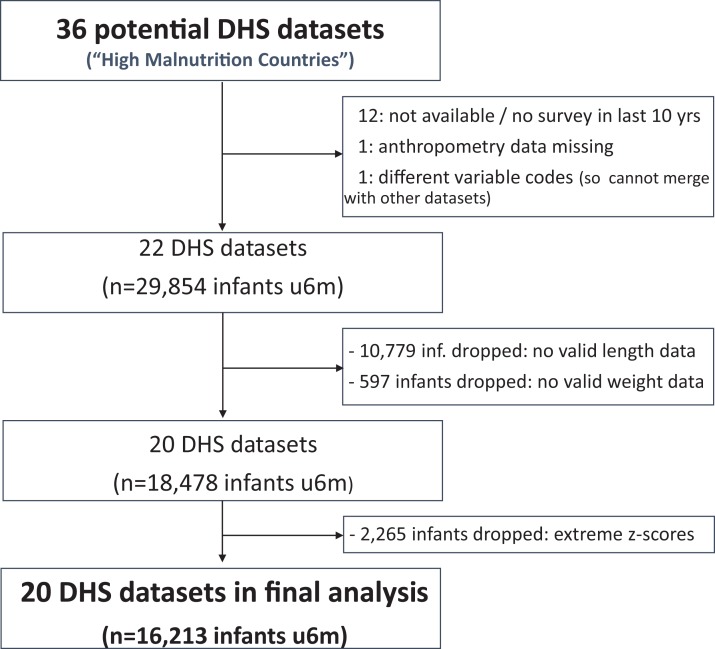
Study flow chart.

Of the 36 ‘high burden’ countries, 20 had relevant data. The flow chart in Fig. 1 summarises how we got to the final study database.

We found 22 eligible DHS datasets with data on 29,854 infants u6m. Excluding those with no length data, the number dropped to 19,075. Eligible infants further dropped to 18,478 after excluding those with no weight data. Of the 18,478 infants u6m remaining (now from 20 countries), 2,265 had out-of-range WLZ, WAZ or LAZ and were thus excluded from further analysis. Of the 16,213 infants u6m included in the final analysis, 8,206 (51%) were boys and 11,352 (70%) lived in a rural area. There were fewest infants in the 0 to <1 month age bracket (1,083 (7%)); 2,706 (17%) in the 1 to <2 month age bracket; then just over 3,000 (19%) in each of the other four age bands u6m. Tables S1A and S1B list and describe the DHS databases that were included in the study and Table S1C summarises the reasons for excluding the 16 others.

Prevalence of severe and moderate wasting and wasting overall is shown in Table 1. There was a wide range in prevalence of both severe wasting (2.1% in Malawi to 13.4% in India) and moderate wasting (3.4% in Burundi to 17.1% in India). Median severe wasting prevalence was 5.6%, median moderate wasting prevalence was 8.1% and median overall wasting prevalence 14.7%.

**Table 1:** Severe infant u6m wasting, moderate infant u6m wasting and overall wasting estimates per country and overall.

Country		Severe wasting		Moderate wasting		Overall wasting	
	*N*	%	95% CI	%	95% CI	%	95% CI
South East Asia Region*							
Bangladesh (2011)	672	5.8	[4.1, 8.3]	9.6	[7.3, 12.6]	15.4	[12.3, 19.2]
India (2005/6)	3,349	13.4	[12.0, 14.9]	17.1	[15.4, 18.9]	30.5	[28.5, 32.5]
Nepal (2011)	215	5.1	[2.6, 10.0]	5.9	[3.2, 10.5]	11	[7.1, 16.6]
**Eastern Mediterranean Region**							
Pakistan (2012/13)	283	6.7	[3.8, 11.5]	8.3	[5.1, 13.3]	15	[10.6, 20.9]
**Western Pacific Region**							
Cambodia (2010)	301	5.3	[3.1, 8.9]	11.1	[7.3, 16.5]	16.4	[11.7, 22.4]
**Africa Region**							
Burkina Faso (2010)	698	11	[8.8, 13.7]	14	[11.5, 16.9]	25	[21.8, 28.5]
Burundi (2010/11)	342	2.6	[1.3, 5.1]	3.4	[1.9, 6.1]	6	[3.9, 9.3]
DRC^†^ (2013/14)	928	4.7	[3.0, 7.1]	7.7	[5.5, 10.5]	12.3	[9.6, 15.6]
Cote d’Ivoire (2011/12)	374	5.9	[3.6, 9.4]	8.8	[5.7, 13.4]	14.7	[10.8, 19.8]
Cameroon (2011)	517	2.8	[1.6, 5.0]	6.9	[4.8, 9.8]	9.7	[7.3, 13.0]
Egypt (2014)	1,210	8	[6.3, 10.2]	6.6	[5.2, 8.4]	14.7	[12.4, 17.2]
Ethiopia (2014)	997	5.4	[3.7, 8.0]	7.8	[5.7, 10.7]	13.3	[10.5, 16.7]
Ghana (2008)	217	6	[3.4, 10.3]	10.1	[6.6, 15.0]	16	[11.7, 21.6]
Kenya (2008/9)	494	4.9	[3.1, 7.8]	4.4	[2.8, 6.8]	9.3	[6.8, 12.7]
Mali (2012/13)	317	9.8	[6.8, 14.0]	8.4	[5.5, 12.7]	18.2	[13.7, 23.9]
Malawi (2010)	350	2.1	[0.9, 4.9]	4.9	[3.0, 7.9]	7	[4.5, 10.6]
Mozambique (2011)	936	4.4	[2.8, 7.0]	6.3	[4.7, 8.4]	10.8	[8.4, 13.7]
Nigeria (2013)	2,457	12.1	[10.5, 13.9]	12	[10.6, 13.7]	24.1	[22.0, 26.4]
Niger (2012)	526	7.7	[4.8, 12.1]	12	[9.3, 15.5]	19.7	[15.6, 24.5]
Zambia (2013/14)	1,030	3.1	[2.2, 4.5]	4.8	[3.5, 6.5]	7.9	[6.3, 10.0]
Total	16,213	10.5	[9.8, 11.4]	13.2	[12.2, 14.2]	23.7	[22.6, 24.9]

**Notes:**

* Regions are as per WHO grouping.

† DRC, Democratic Republic of the Congo.

Table 2 is an unadjusted analysis exploring the association between household characteristics and infant u6m wasting. There is a statistically significant greater odds of wasting among infants u6m whose families have no toilet compared with those who have improved toilets. Non-improved toilets are, however, associated with a significantly lower risk of wasting. We did not have data on whether the toilet was shared or unique to one household. Rural or urban residence, water source, time to fetch water and livestock ownership showed no clear associations. ORs for wealth quintiles show the expected gradient whereby compared to a middle quintile reference, infants of the poorest families are more likely to be wasted; those from the richest families are less likely to be wasted. Statistically significant differences (*p* < 0.05) were only observed at the two extremes: poorest and richest vs. middle quintile. A test of trend for wealth index was also statistically significant: for each unit increase in wealth, the odds of infant wasting decreased by 9% (95% CI [13–5%] reduction), *p* < 0.001.

**Table 2:** Household characteristics and their association with infant u6m wasting.

Household characteristic	OR	95% CI		*p*-value
Residence (*N* = 16,213)				
Rural (vs. *urban reference*)	1.09	0.95	1.26	0.21
Water source (*N* = 14,982)				
Non-improved (vs. *improved ref*).	0.86	0.75	1.00	0.05
Time to fetch water (*N* = 14,878)				
Source on premises	1 (ref)	–	–	–
Source at ≤ 30 min	1.04	0.90	1.19	0.64
Source at >30 min	0.83	0.67	1.01	0.07
Toilet type (*N* = 14,961)				
Improved	1 (ref)	–	–	–
Non-improved	0.68	0.57	0.81	<0.001**
No toilet	1.55	1.34	1.78	<0.001**
Wealth index (*N* = 16,213)				
Poorest	1.22	1.01	1.48	0.04*
Poorer	1.05	0.86	1.28	0.62
Middle	1 (ref)	–	–	–
Richer	0.95	0.78	1.16	0.63
Richest	0.78	0.63	0.95	0.02*
Own livestock (16,211)				
Has animals (vs. *none ref*)	0.99	0.87	1.14	0.93

**Notes:**

* *p* < 0.05.

** *p* < 0.01.

The same analyses subdividing the outcome into moderate and severe wasting is shown in Table S2; the overall pattern of these observations is similar.

Table 3 explores maternal characteristics and relationships and their association with infant u6m wasting. Results are also presented adjusted for infant age group, sex and socioeconomic status. Maternal age shows no statistically significant associations but maternal anthropometry does. Low maternal body mass index (BMI) (BMI < 18.5) is associated with statistically significantly greater odds of infant u6m wasting in both unadjusted and adjusted analysis. In contrast, high maternal BMI is associated with reduced odds of infant u6m wasting—though only at very high BMI (>30) is this statistically significant. Mothers who are stunted (height < 145 cm) are also statistically significantly more likely to have a wasted infant u6m on unadjusted analysis; after adjusting, this association weakens to a statistically borderline *p*-value of 0.05. Compared to mothers with no education, those who have attended school are less likely to have a wasted infant u6m compared to those with no education. A test of trend of wasting vs. level of education is statistically significant (*p* < 0.001) on unadjusted analysis but not on adjusted analysis (*p* = 0.32). After adjusting, only primary education vs. no education is statistically associated.

**Table 3:** Maternal characteristics and their association with infant u6m wasting.

	Unadjusted				Adjusted^†^			
	OR	95% CI		*p*-value	OR	95% CI		*p*-value
Age (*N* = 16,213)								
<20 years	1 (ref)	–	–	–	1 (ref)	–	–	–
≥20 and <35	0.99	0.81	1.20	0.89	1.00	0.82	1.22	0.99
≥35	0.98	0.76	1.26	0.88	1.02	0.78	1.32	0.91
BMI (*N* = 16,213)								
<18.5 (low)	1.61	1.37	1.89	<0.001**	1.53	1.29	1.80	<0.001**
≥18.5 and <25.0 (normal)	1 (ref)	–	–	–	1 (ref)	–	–	–
≥25.0 and <30.0 (high)	0.78	0.65	0.95	0.01*	0.84	0.69	1.03	0.09
≥30.0 (very high)	0.48	0.36	0.63	<0.001**	0.49	0.36	0.65	<0.001**
Height (*N* = 16,213)								
<145 cm (stunted) (vs. *≥145 cm ref*)	1.36	1.07	1.73	0.01*	1.26	1.00	1.61	0.05
Education (*N* = 16,211)								
No education	1 (ref)	–	–	–	1 (ref)	–	–	–
Primary	0.64	0.54	0.76	<0.001**	0.71	0.60	0.85	<0.001**
Secondary	0.86	0.74	1.00	0.05	0.97	0.83	1.15	0.76
Higher	0.64	0.49	0.84	<0.01**	0.80	0.58	1.10	0.15
Working (*N* = 16,208)								
Working (vs. *not working ref*)	0.82	0.72	0.94	<0.01**	0.84	0.74	0.96	0.01
Maternal relationships (*N* = 16,213)								
In union (vs. *not in union ref*)	2.09	1.67	2.62	<0.001**	1.89	1.51	2.37	<0.001**
Who decides about health issues (*N* = 15,252)								
Respondent	1 (ref)	–	–	–	1 (ref)	–	–	–
Respondent + husband	1.09	0.88	1.34	0.43	1.13	0.92	1.39	0.25
Husband	1.21	0.99	1.48	0.06	1.21	0.99	1.48	0.06
Other	1.49	1.11	2.00	<0.01**	1.42	1.05	1.92	0.02*
Violence (vs. no violence reference)								
Any violence (*N* = 8,623)	1.28	1.08	1.52	<0.01**	1.26	1.06	1.50	0.01*
Emotional (*N* = 8,382)	1.06	0.84	1.34	0.63	1.09	0.86	1.39	0.49
Physical (*N* = 8,381)	1.34	1.12	1.62	<0.01**	1.30	1.08	1.57	<0.01**
Severe Phys. (*N = 8,379*)	1.07	0.80	1.43	0.63	1.05	0.78	1.41	0.76
Sexual (*N* = 8,379)	1.32	1.00	1.73	0.05	1.28	0.96	1.69	0.09

**Notes:**

* *p* < 0.05.

** *p* < 0.01.

† Adjusted for infant age group, sex and socio-economic status.

Compared with non-working mothers, those in work are significantly less likely to have wasted infants u6m; this association remains statistically significant even after adjusting.

A mother being in a relationship (‘in union’) is also associated with statistically greater odds of having a wasted infant u6m: both on unadjusted and adjusted analysis. Whenever it is someone other than the mother/carer who decides about health issues in the household, the odds of having a wasted infant u6m are increased; the association is of borderline statistical significance (*p* = 0.06) when it is the husband deciding and strongly significant (*p* = 0.02) when anyone else in the household decides. Reported experience of violence is also a risk factor with statistically significant associations with wasting. Physical violence is the subtype of domestic violence with statistically significant associations.

Table S3 presents the same variables with wasting subdivided into severe and moderate. Again, the general patterns of observations are similar.

Table 4 shows infant u6m characteristics and their association with infant u6m wasting. Infant age group, infant sex, birth spacing, birth order and history of previous infant death are not associated with either increased or decreased odds of infant u6m wasting. Regarding antenatal practice, a history of the recommended minimum four antenatal visits by a skilled provider is associated with statistically significant decreased odds of infant u6m wasting. Birth at home rather than in a health facility is associated with statistically greater odds of wasting. Birth by caesarean section is associated with statistically significant decreased odds of wasting, even after adjusting. Test of trend reveals statistically significant association between reported size at birth and later wasting: aOR 1.11, 95% CI [1.03–1.19], *p* = 0.004. The only statistically significant individual birth size strata is not statistically associated with later wasting; infants who were reportedly large at birth being less likely to be wasted and those who were small at birth more likely to be wasted. A statistically significant association is only observed for those infants u6m who were reported to be smaller than average at birth, they are significantly more likely to be wasted, albeit with an OR of only 1.32 (95% CI [1.10–1.58], *p* < 0.01).

**Table 4:** Infant characteristics and their association with infant u6m wasting (WLZ <−2).

		Unadjusted				Adjusted^†^			
		OR	95% CI		*p*-value	OR	95% CI		*p*-value
Child age group (*N* = 16,213)	*0–2 months*	1 (*ref*)	–	–	–				
	*3–5 months*	0.95	0.83	1.08	0.45	0.91	0.69	1.19	0.48
Child sex (*N* = 16,213)	*Female* (vs. *male ref*)	1.01	0.89	1.16	0.83	1.00	0.88	1.14	1.00
Birth order (*N* = 16,213)	1	1 (*ref*)	–	–	–				
	2	1.04	0.87	1.25	0.66	1.02	0.85	1.23	0.80
	3	1.00	0.82	1.23	0.97	0.98	0.80	1.21	0.87
	4	1.06	0.90	1.27	0.48	1.00	0.83	1.22	0.97
Birth spacing (*N* = 12,373)	≤24 months	1 (*ref*)	–	–	–				
	>24 month	1.20	1.00	1.44	0.05	1.18	0.98	1.41	0.08
Previous child death (*N* = 16,213)	None	1 (*ref*)	–	–	–				
	One	0.99	0.82	1.19	0.90	0.95	0.78	1.15	0.58
	Two or over two	1.08	0.85	1.36	0.53	1.00	0.79	1.28	0.97
Antenatal care (ANC) and birth history									
Appropriate ANC 4+ visits by skilled provider (vs. *not ref*) (*N* = 15,908)		0.72	0.64	0.82	<0.001**	0.77	0.67	0.89	<0.001**
Born at home (vs. *facility ref*) (*N* = 16,099)		1.41	1.23	1.60	<0.001**	1.30	1.12	1.51	<0.01**
Born by C-section (vs. *not ref*) (*N* = 16,181)		0.71	0.57	0.88	<0.01**	0.73	0.58	0.92	<0.01**
^‡^Size at birth (*N* = 16,035)									
	Largest	0.83	0.62	1.09	0.18	0.89	0.67	1.18	0.41
	Larger than av.	0.92	0.76	1.10	0.33	0.96	0.80	1.15	0.64
	Average	1 (*ref*)	–	–	–	*1* (*ref*)	–	–	–
	Smaller than av.	1.35	1.13	1.61	<0.01**	1.32	1.10	1.58	<0.01**
	Very small	1.23	0.98	1.56	0.08	1.19	0.94	1.50	0.15
Postnatal care (*N* = 11,258) *Does not include phase 5 countries: Ghana, India, Kenya, Cambodia, Malawi*									
	Yes (vs. *no ref*)	0.84	0.73	0.97	0.02*	0.90	0.77	1.05	0.17
Breastfeeding (BF)-related:									
*Started BF* (*N = 15,786*)*:*	Within 1 h	1 (*ref*)	–	–	–				
	Within 1 day	1.36	1.17	1.57	<0.001**	1.31	1.13	1.51	<0.001**
	>1 day	1.85	1.55	2.21	<0.001**	1.65	1.38	1.98	<0.001**
*Fed anything before BF* (*N = 16,213*), (vs. *not*)		1.46	1.29	1.66	<0.001**	1.34	1.18	1.53	<0.001**
*Ever breastfed* (*N = 16,204*)	(vs. *no ref*)	0.38	0.21	0.70	<0.01**	0.36	0.20	0.65	<0.01**
*Currently BF* (*N = 16,213*)	“”	0.65	0.44	0.97	0.04*	0.62	0.42	0.91	0.02*
*Exclusively BF* (*16,149*)	“”	0.89	0.78	1.03	0.11	0.84	0.73	0.97	0.02*
*Predominant BF* (*N = 16,071*)	“”	1.05	0.91	1.21	0.51	0.95	0.82	1.10	0.51
*Bottle fed* (*N = 16,201*)	“”	0.99	0.82	1.20	0.94	1.10	0.90	1.33	0.35
Vaccine-related									
*Has vacc. card* (*N = 16,210*) (vs. *no ref*)		0.80	0.70	0.91	<0.01**	0.86	0.74	0.99	0.03*
*Has BCG card* (*N = 16,196*) (vs. *no ref*)		0.79	0.69	0.90	<0.001**	0.85	0.74	0.97	0.02*
*Timely vac. on card* (*DTP, Polio*) (*N = 16,150*)		0.80	0.70	0.92	<0.01**	0.86	0.75	0.99	0.03*
Recent illness episodes (in last 2 weeks)									
Fever (*N* = 16,197)	(vs. *no ref*)	1.06	0.88	1.26	0.54	1.06	0.89	1.28	0.50
Cough (*N* = 16,182)	“”	0.96	0.82	1.13	0.63	0.97	0.83	1.15	0.76
Diarrhoea (*N* = 16,200)	“”	1.33	1.10	1.63	<0.001**	1.37	1.12	1.67	<0.01**

**Notes:**

* *p* < 0.01.

** *p* < 0.001.

† Adjusted for infant age group, sex and socio-economic status.

‡ This is size as reported by the carer rather than measured size.

Numerous breastfeeding (BF) related indicators are statistically associated with infant u6m wasting. Late initiation of BF and a history of a prelacteal feed are both statistically associated with greater odds and adjusted odds of infant u6m wasting; ever breastfed, currently BF and exclusively BF are associated with significantly decreased odds of wasting.

A history of following vaccine-related recommendations is associated with statistically significantly decreased odds of infant u6m wasting. This includes having a vaccine card and having recorded timely DTP (diphtheria, pertussis and tetanus) and polio vaccine.

Finally, a 2-week illness history of fever and cough were not associated with infant u6m wasting, but those with a reported history of diarrhoea within the last 2 weeks were significantly more likely to be wasted.

Table S4 presents the same variables with wasting subdivided into severe and moderate. As before, the general patterns of observations are similar.

## Discussion

In this paper we explored a large number of factors which could plausibly increase or decrease the risk of acute wasting in infants u6m and found numerous statistically significant associations. These spanned all three of our conceptual categories: household factors; maternal factors; infant factors. For the majority of these, the direction of association is biologically or socially plausible and a probable mechanism can easily be understood. Noteworthy factors associated with increased odds of infant u6m wasting included: poverty; maternal malnutrition (especially low BMI); uneducated mother; mother disempowered (experiences violence; does not make decisions about health issues; does not engage with health services such as antenatal care, does not give birth in a health facility); reported small infant size at birth (a maker of low birth weight, which has previously reported associations with later growth and health (Blanc & Wardlaw, 2005; Martin et al., 2017; Villar et al., 2018)); poor early breastfeeding practices (delayed initiation of BF; prelacteal feed); recent history of diarrhoea. ‘Protective’ factors associated with significantly decreased odds of infant u6m wasting included: wealthier family; normal (and even high) maternal BMI; mother in work; positive BF practices (ever breastfed currently BF, exclusively BF); engagement with vaccination services. Notable negatives include lack of an association with infant sex, birth order and birth spacing. To our knowledge, this is the first paper to attempt such detailed analysis focused on wasting in the infant u6m age group alone.

We are fully aware of the limitations of our work:

Firstly, we emphasise that any associations observed in a cross-sectional study such as ours cannot prove causation, however plausible they may be. Special care must be taken regarding possible directions of association (e.g. reverse causality: rather than a non-exclusively breastfed infant becoming malnourished as a consequence, a malnourished exclusively breastfed infant may be introduced to other foods/fluids in an attempt to stop further deterioration). Bradford-Hill criteria can help when considering possible causality of individual factors (Potischman & Weed, 1999; Weed, 1997). These include: consistency with other literature, biological plausibility and coherence of evidence. For instance, the benefits of breastfeeding have been extensively described in many other populations and many of its mechanisms elucidated (Oddy, 2002; Victora et al., 2016; Perez-Escamilla, Martinez & Segura-Perez, 2016). Other observations consistent with existing literature looking at malnutrition in wider child age groups include: poverty, poor home environment and poorly educated mother (long recognised as a cause of malnutrition) (Black et al., 2008); domestic violence (shown to affect growth in all children aged <5 years) (Rico et al., 2011; Yount, DiGirolamo & Ramakrishnan, 2011; Ziaei, Naved & Ekstrom, 2012); short maternal stature—associated with risk of small for gestational age and preterm birth in a large meta-analysis (Kozuki et al., 2015; Wrottesley, Lamper & Pisa, 2016). Lack of association with birth spacing was a key negative observation in our study: this is also consistent with a 2007 review which found variable effects of birth spacing on child nutrition in different studies and settings (Dewey & Cohen, 2007).

Strength of association is another important Bradford-Hill criterion for causality. Even when statistically significant, most of our ORs are not far from 1. No one factor alone has a very high OR, as might be the case if it played a dominant causal role. Many weak associations as in our paper are not uncommon in nutritional epidemiology (Potischman & Weed, 1999).

Second, we are conscious of having conducted multiple analyses and therefore expect some statistically significant associations by chance alone. Again, Bradford-Hill criteria can help interpret both expected and some unexpected observations (e.g. we expected that there would be less rather than more wasting among infants in homes with improved toilets. Perhaps this observation is due to chance alone. It could also be due to unexplained confounding such as improved toilets more likely to be shared and/or poorly maintained).

Third, we acknowledge that our dataset was not designed for looking at infants u6m alone. Consistent with other studies (Grijalva-Eternod et al., 2016) we found lots of exclusions due to missing anthropometric data, notably length. Of those we could look at, some observations may just reflect a small sample size. This is also the reason why we deliberately did not attempt more complex modelling or statistical adjustments. We felt that this might increase the risk of over-interpretation of the data: better to use our results to shape more definitive future studies.

Fourth, we are conscious of an active debate around case definitions of acute malnutrition in infants u6m. In this paper, we focus on wasting as defined by low weight-for-length because this is currently recommended by WHO (2013) but this measure has limitations (e.g. WLZ cannot be calculated where length is <45 cm; WHO growth standards as used here do not take into account that some infants u6m are small due to prematurity but may be appropriately sized for gestational age). With different measures of malnutrition as the outcome variable, different associations or different strengths of association might be observed. Future analyses focused on low WAZ and/or LAZ as the outcome would be valuable. Looking forwards, there is increasing interest in other ways of identifying nutritionally vulnerable infants u6m, including by MUAC (Angood et al., 2015; Mwangome et al., 2012a; 2012b), although this is currently not measured in DHS. Another DHS limitation is that not all relevant factors are explored; for example, due to lack of data, it was not possible to explore the role of maternal mental health and its impact on infant/child nutrition (Stewart, 2007; Stewart et al., 2008). This should be considered in future work and programme design; peer support and women’s groups have proven success for other mother/child health outcomes and could be adapted to address infant nutrition (Prost et al., 2013; Yousafzai et al., 2014).

For these reasons, we emphasise that our data is hypothesis generating. We intended to inform and advance current programmes and practices in this area, especially to give background for future research which can more definitively answer key questions arising. Balancing the limitations, we believe that there are many strengths of our analysis and several messages arising which are important and relevant to both practitioners and researchers.

First, we found many factors associated with infant u6m wasting. This supports calls for broader and more integrated prevention and treatment packages (Kerac et al., 2015; ENN/UCL/ACF, 2010). The treatment of SAM and MAM in older children is focused around ready-to-use foods as the core intervention. These are not appropriate for infants u6m and there is no equivalent ‘magic bullet’ which alone is likely to dominate treatment of infant u6m malnutrition. Breastfeeding support is clearly necessary and will be core to any future intervention package: subcomponents should include issues like encouraging early initiation of BF and avoidance of prelacteal feeds. However, this it alone is unlikely to be sufficient. The many other risk factors we observed also need to be addressed. Some lead to clear and relatively straightforward interventions (e.g. encourage greater engagement with and enable access to antenatal care services and continue emphasis on vaccinations). Others require deeper societal change (e.g. poverty reduction, continued improvements in education and female education, in particular). Others are arguably important whatever the association with infant u6m wasting (e.g. a safe and hygienic environment through good Water and Sanitation for Health (WASH) facilities is a basic human right (Dangour et al., 2013; Gine-Garriga et al., 2017); even had we not observed an association between domestic violence and infant u6m wasting, there are many other moral and ethical reasons why it should be addressed within a maternal-support strategy). Others still need further exploration in specifically designed future studies to determine if causal and if so via which mechanism (e.g. the observation about C-Section being associated with less wasting could just be due to chance or might be a marker of better access to healthcare in general which cannot be directly measured/assessed from our current dataset).

Second, we also found that not all variables examined were in fact associated with wasting (e.g. birth order—it is plausible that first infants are most at risk due to maternal inexperience, but this turns out not to be the case with all potentially at risk and all mothers potentially needing support. This could be because maternal resources are depleted with subsequent pregnancies). Excluding some variables in this way thus provides greater focus for future research: rather than investigate all possible risk factors as we have done, others can investigate a smaller number of risk factors in more detail, to better understand how they impact infant u6m malnutrition and how they might play a role in future intervention strategies.

Third, our findings are helpful in shaping clinical assessment tools. One such tool is the ‘C-MAMI’ (Community Management of Acute Malnutrition) tool which takes a similarly broad approach to assessing and managing infants <6 m (Emergency Nutrition Network (ENN), 2016). At core it focuses on breastfeeding support but it also considers wider issues such as carer employment and sources of social support: addressing these might do much to better empower mothers and thus directly benefit both them and their infants. By identifying key risk factors, we hope to inform future versions of this tool, as well as intervention studies to test the tool.

Finally, our focus on infants u6m raises their profile. Other papers have looked at infant malnutrition in general but ours adds to the literature by focusing just on wasting and just on infants u6m, a very specific subgroup who, according to WHO (2013) and others (as per intro to this paper) are often overlooked. We hope that future research and surveys likewise focus on this group alone and/or are adequately powered to conduct robust subgroup analysis—rather than lumping data with that of older children and others. This will facilitate better future understanding of malnutrition in this age group.

There is much scope for future research. Although our results are based on a large number of countries and thus broadly generalizable, individual countries planning their services may want to consider more local data from their own DHS or similar. Above all, however, there is a need for prospective research and especially for intervention studies to address infant u6m malnutrition. Our results could inform sample size calculations for such projects, giving investigators an idea of possible effect sizes. Most importantly, targeting single risk factors alone is unlikely to have the optimal impact, intervention ‘packages’ delivering multiple strands of care that consider infant, maternal and household factors are likely to be more successful, potentially even synergistic in their effects. For instance, breastfeeding support should be combined with programmes addressing gender inequalities and empowering women and girls (Kraft et al., 2014; Mandal, Muralidharan & Pappa, 2017; Orton et al., 2016; Taukobong et al., 2016). ‘Positive deviance/hearth’ approaches also have potential future roles this area (Bisits Bullen, 2011).

## Conclusions

Infant u6m wasting is a complex, multifactorial problem associated with numerous risk factors encompassing household, maternal and infant characteristics. Many of those which we identified in our dataset are biologically plausible and/or socially important and could be included in assessment tools and intervention strategies to help nutritionally vulnerable infants u6m. Evidence-based breastfeeding support is vital and needs to be further strengthened in many settings (especially in communities without safe water and during emergencies). However, this alone may not be sufficient. Packages of care should also seek to support and empower mothers. Our data relating to antenatal care and early breastfeeding practices point to possible benefits of more proactive/early interventions that seek to prevent as well as treat acute malnutrition, and the need for collaboration between sectors (e.g. health, mental health, social, WASH, nutrition) and service delivery providers.

There is an urgent need to test these insights from observational work in future intervention studies. Our results will help design such studies, focus resources and maximise chances of successful identification and management of nutritionally vulnerable infants u6m.

## Supplemental Information

10.7717/peerj.5848/supp-1 Supplemental Information 1 Strobe Checklist.

10.7717/peerj.5848/supp-2 Supplemental Information 2 Characteristics of the database–Countries.

10.7717/peerj.5848/supp-3 Supplemental Information 3 Characteristics of the database–infants.

10.7717/peerj.5848/supp-4 Supplemental Information 4 Countries not included and reason for exclusion.

10.7717/peerj.5848/supp-5 Supplemental Information 5 Household characteristics and their association with infant u6m wasting–subdivided into severe and moderate wasting.

10.7717/peerj.5848/supp-6 Supplemental Information 6 Maternal characteristics and their association with infant u6m wasting–subdivided into severe and moderate wasting. *P<0.01, **P<0.001.
†Adjusted for infant age group, sex and socio-economic status.

10.7717/peerj.5848/supp-7 Supplemental Information 7 Infant characteristics and their association with infant u6m wasting–subdivided into severe and moderate wasting. *P<0.01, **P<0.001.
‡This is size as reported by the carer rather than measured size.
†Adjusted for infant age group, sex and socio-economic status.

## Abbreviations

aOR: Adjusted odds ratio
BF: Breastfeeding
BMI: Body Mass Index
CHNRI: Child Health and Nutrition Research Initiative
DHS: Demographic and Health Survey
DPT: Diphtheria, pertussis and tetanus
HAZ: Height-for-age *z*-score
LAZ: Length-for-age *z*-score
MAM: Moderate Acute Malnutrition
MUAC: Mid Upper Arm Circumference
OR: Odds Ratio
SAM: Severe Acute Malnutrition
U5: Under 5 years
u6m: Under 6 months
WASH: Water and Sanitation for Health
WAZ: Weight-for-age *z*-score
WLZ: Weight-for-length *z*-score
WHO: World Health Organization.

## Additional Information and Declarations

### Data Availability

The following information was supplied regarding data availability: All the DHS datasets containing all the variables presented in our analysis are freely available to download online via registration with DHS: https://dhsprogram.com/data/Access-Instructions.cfm.

## References

[ref-5] Angood C, McGrath M, Mehta S, Mwangome M, Lung’aho M, Roberfroid D, Perry A, Wilkinson C, Israel A-D, Bizouerne C, Haider R, Seal A, Berkley JA, Kerac M, Collaborators MWG (2015). Research priorities to improve the management of acute malnutrition in infants aged less than six months (MAMI). PLOS Medicine.

[ref-6] Bhutta ZA (2013). Early nutrition and adult outcomes: pieces of the puzzle. Lancet.

[ref-7] Bisits Bullen PA (2011). The positive deviance/hearth approach to reducing child malnutrition: systematic review. Tropical Medicine & International Health.

[ref-8] Black RE, Allen LH, Bhutta ZA, Caulfield LE, De Onis M, Ezzati M, Mathers C, Rivera J (2008). Maternal and child undernutrition: global and regional exposures and health consequences. Lancet.

[ref-9] Black RE, Victora CG, Walker SP, Bhutta ZA, Christian P, De Onis M, Ezzati M, Grantham-McGregor S, Katz J, Martorell R, Uauy R, Maternal and Child Nutrition Study Group (2013). Maternal and child undernutrition and overweight in low-income and middle-income countries. Lancet.

[ref-10] Blanc AK, Wardlaw T (2005). Monitoring low birth weight: an evaluation of international estimates and an updated estimation procedure. Bull World Health Organ.

[ref-11] Crowe S, Seal A, Grijalva-Eternod C, Kerac M (2014). Effect of nutrition survey ‘cleaning criteria’ on estimates of malnutrition prevalence and disease burden: secondary data analysis. PeerJ.

[ref-12] Dangour AD, Watson L, Cumming O, Boisson S, Che Y, Velleman Y, Cavill S, Allen E, Uauy R (2013). Interventions to improve water quality and supply, sanitation and hygiene practices, and their effects on the nutritional status of children. Cochrane Database Systematic Reviews.

[ref-13] Dewey KG, Cohen RJ (2007). Does birth spacing affect maternal or child nutritional status? A systematic literature review. Maternal & Child Nutrition.

[ref-1] Demographic and Health Surveys (DHS) (2017a). Accessing data. https://dhsprogram.com/data/new-user-registration.cfm.

[ref-14] Demographic and Health Surveys (DHS) (2017b). Data variables and definitions. http://dhsprogram.com/data/data-variables-and-definitions.cfm.

[ref-15] Demographic and Health Surveys (DHS) (2017c). DHS methodology: survey process, questionnaires, GIS, biomarkers, protecting the privacy of DHS survey respondents. http://dhsprogram.com/What-We-Do/methodology.cfm.

[ref-16] Demographic and Health Surveys (DHS) (2017d). DHS program: who we are. http://dhsprogram.com/Who-We-Are/About-Us.cfm.

[ref-17] Demographic and Health Surveys (DHS) (2017e). DHS wealth index construction. http://www.dhsprogram.com/topics/wealth-index/Wealth-Index-Construction.cfm.

[ref-4] Emergency Nutrition Network (ENN) (2016). A simple IMCI-sytle tool for assessing, identifing/classifying and managing uncomplicated acute malnutrition in infants < 6 months of age in the community–the “c-MAMI” tool. http://www.ennonline.net/c-mami.

[ref-18] ENN/UCL/ACF (2010). Management of acute malnutrition in infants (MAMI) project. Emergency nutrition network, UCL Centre for International Health & Development, action contre la faim. http://www.ennonline.net/mamitechnicalreview.

[ref-19] Frison S, Checchi F, Kerac M (2015). Omitting edema measurement: how much acute malnutrition are we missing?. American Journal of Clinical Nutrition.

[ref-20] Gine-Garriga R, Flores-Baquero O, Jimenez-Fdez De Palencia A, Perez-Foguet A (2017). Monitoring sanitation and hygiene in the 2030 agenda for sustainable development: a review through the lens of human rights. Science of the Total Environment.

[ref-3] Global Strategy for Women’s, Children’s and Adolescents’ Health (2016). The Global Strategy for Women’s, Children’s and Adolescents’ Health (2016–2030). http://globalstrategy.everywomaneverychild.org/pdf/EWEC_globalstrategyreport_200915_FINAL_WEB.pdf.

[ref-21] Gluckman PD, Hanson MA, Pinal C (2005). The developmental origins of adult disease. Maternal & Child Nutrition.

[ref-22] Grijalva-Eternod CS, Kerac M, McGrath M, Wilkinson C, Hirsch JC, Delchevalerie P, Seal AJ (2016). Admission profile and discharge outcomes for infants aged less than 6 months admitted to inpatient therapeutic care in 10 countries. A secondary data analysis. Maternal & Child Nutrition.

[ref-23] Hawkes C, Popkin BM (2015). Can the sustainable development goals reduce the burden of nutrition-related non-communicable diseases without truly addressing major food system reforms?. BMC Medicine.

[ref-24] ICF International (2012). Sampling and household listing manual: demographic and health surveys methodology. ICF international calverton, Maryland USA. https://dhsprogram.com/pubs/pdf/DHSM4/DHS6_Sampling_Manual_Sept2012_DHSM4.pdf.

[ref-25] Kerac M, Blencowe H, Grijalva-Eternod C, McGrath M, Shoham J, Cole TJ, Seal A (2011). Prevalence of wasting among under 6-month-old infants in developing countries and implications of new case definitions using WHO growth standards: a secondary data analysis. Archives of Disease in Childhood.

[ref-26] Kerac M, Mwangome M, McGrath M, Haider R, Berkley JA (2015). Management of acute malnutrition in infants aged under 6 months (MAMI): current issues and future directions in policy and research. Food and Nutrition Bulletin.

[ref-27] Kozuki N, Katz J, Lee AC, Vogel JP, Silveira MF, Sania A, Stevens GA, Cousens S, Caulfield LE, Christian P, Huybregts L, Roberfroid D, Schmiegelow C, Adair LS, Barros FC, Cowan M, Fawzi W, Kolsteren P, Merialdi M, Mongkolchati A, Saville N, Victora CG, Bhutta ZA, Blencowe H, Ezzati M, Lawn JE, Black RE, Child Health Epidemiology Reference Group Small-for-Gestational-Age/Preterm Birth Working Group (2015). Short maternal stature increases risk of small-for-gestational-age and preterm births in low- and middle-income countries: individual participant data meta-analysis and population attributable fraction. Journal of Nutrition.

[ref-28] Kraft JM, Wilkins KG, Morales GJ, Widyono M, Middlestadt SE (2014). An evidence review of gender-integrated interventions in reproductive and maternal-child health. Journal of Health Communication.

[ref-29] Mandal M, Muralidharan A, Pappa S (2017). A review of measures of women’s empowerment and related gender constructs in family planning and maternal health program evaluations in low- and middle-income countries. BMC Pregnancy and Childbirth.

[ref-30] Martin A, Connelly A, Bland RM, Reilly JJ (2017). Health impact of catch-up growth in low-birth weight infants: systematic review, evidence appraisal, and meta-analysis. Maternal & Child Nutrition.

[ref-31] Mwangome MK, Fegan G, Fulford T, Prentice AM, Berkley JA (2012a). Mid-upper arm circumference at age of routine infant vaccination to identify infants at elevated risk of death: a retrospective cohort study in the Gambia. Bulletin of the World Health Organization.

[ref-32] Mwangome MK, Fegan G, Mbunya R, Prentice AM, Berkley JA (2012b). Reliability and accuracy of anthropometry performed by community health workers among infants under 6 months in rural Kenya. Tropical Medicine & International Health.

[ref-33] No Wasted Lives (2018). No Wasted Lives. https://www.nowastedlives.org/.

[ref-34] Oddy WH (2002). Long-term health outcomes and mechanisms associated with breastfeeding. Expert Review of Pharmacoeconomics & Outcomes Research.

[ref-35] Orton L, Pennington A, Nayak S, Sowden A, White M, Whitehead M (2016). Group-based microfinance for collective empowerment: a systematic review of health impacts. Bulletin of the World Health Organization.

[ref-36] Perez-Escamilla R, Martinez JL, Segura-Perez S (2016). Impact of the baby-friendly hospital initiative on breastfeeding and child health outcomes: a systematic review. Maternal & Child Nutrition.

[ref-37] Potischman N, Weed DL (1999). Causal criteria in nutritional epidemiology. American Journal of Clinical Nutrition.

[ref-38] Prost A, Colbourn T, Seward N, Azad K, Coomarasamy A, Copas A, Houweling TA, Fottrell E, Kuddus A, Lewycka S, MacArthur C, Manandhar D, Morrison J, Mwansambo C, Nair N, Nambiar B, Osrin D, Pagel C, Phiri T, Pulkki-Brannstrom A-M, Rosato M, Skordis-Worrall J, Saville N, More NS, Shrestha B, Tripathy P, Wilson A, Costello A (2013). Women’s groups practising participatory learning and action to improve maternal and newborn health in low-resource settings: a systematic review and meta-analysis. Lancet.

[ref-39] Rico E, Fenn B, Abramsky T, Watts C (2011). Associations between maternal experiences of intimate partner violence and child nutrition and mortality: findings from Demographic and Health Surveys in Egypt, Honduras, Kenya, Malawi and Rwanda. Journal of Epidemiology & Community Health.

[ref-40] Stewart RC (2007). Maternal depression and infant growth? A review of recent evidence. Maternal & Child Nutrition.

[ref-41] Stewart RC, Umar E, Kauye F, Bunn J, Vokhiwa M, Fitzgerald M, Tomenson B, Rahman A, Creed F (2008). Maternal common mental disorder and infant growth a cross-sectional study from Malawi. Maternal & Child Nutrition.

[ref-42] Taukobong HF, Kincaid MM, Levy JK, Bloom SS, Platt JL, Henry SK, Darmstadt GL (2016). Does addressing gender inequalities and empowering women and girls improve health and development programme outcomes?. Health Policy and Planning.

[ref-43] Tomkins A (2000). Malnutrition, morbidity and mortality in children and their mothers. Proceedings of the Nutrition Society.

[ref-2] United Nations (2015). Sustainable development goals. https://sustainabledevelopment.un.org/?menu=1300.

[ref-44] Victora CG, Adair L, Fall C, Hallal PC, Martorell R, Richter L, Sachdev HS (2008). Maternal and child undernutrition: consequences for adult health and human capital. Lancet.

[ref-45] Victora CG, Bahl R, Barros AJD, Franca GVA, Horton S, Krasevec J, Murch S, Sankar MJ, Walker N, Rollins NC, Lancet Breastfeeding Series Group (2016). Breastfeeding in the 21st century: epidemiology, mechanisms, and lifelong effect. Lancet.

[ref-46] Villar J, Giuliani F, Barros F, Roggero P, Coronado Zarco IA, Rego MAS, Ochieng R, Gianni ML, Rao S, Lambert A, Ryumina I, Britto C, Chawla D, Cheikh Ismail L, Ali SR, Hirst J, Teji JS, Abawi K, Asibey J, Agyeman-Duah J, McCormick K, Bertino E, Papageorghiou AT, Figueras-Aloy J, Bhutta Z, Kennedy S (2018). Monitoring the postnatal growth of preterm infants: a paradigm change. Pediatrics.

[ref-47] Weed DL (1997). On the use of causal criteria. International Journal of Epidemiology.

[ref-48] World Health Organization (WHO) (2011). WHO Anthro (version 3.2.2, January 2011) and macros. http://www.who.int/childgrowth/software/en/.

[ref-49] World Health Organization (WHO) (2013). Updates on the management of severe acute malnutrition in infants and children (Guideline). http://www.who.int/nutrition/publications/guidelines/updates_management_SAM_infantandchildren/en/index.html.

[ref-51] World Health Organization (WHO) (2017). The WHO child growth standards. http://www.who.int/childgrowth/standards/en/.

[ref-50] WHO, UNICEF (2009). WHO child growth standards and the identification of severe acute malnutrition in infants and children. (2009) A joint statement by the World Health Organization and the United Nations Children’s Fund. http://www.who.int/nutrition/publications/severemalnutrition/9789241598163/en/index.html.

[ref-52] Wrottesley SV, Lamper C, Pisa PT (2016). Review of the importance of nutrition during the first 1000 days: maternal nutritional status and its associations with fetal growth and birth, neonatal and infant outcomes among African women. Journal of Developmental Origins of Health and Disease.

[ref-53] Yount KM, DiGirolamo AM, Ramakrishnan U (2011). Impacts of domestic violence on child growth and nutrition: a conceptual review of the pathways of influence. Social Science & Medicine.

[ref-54] Yousafzai AK, Rasheed MA, Rizvi A, Armstrong R, Bhutta ZA (2014). Effect of integrated responsive stimulation and nutrition interventions in the lady health worker programme in Pakistan on child development, growth, and health outcomes: a cluster-randomised factorial effectiveness trial. Lancet.

[ref-55] Ziaei S, Naved RT, Ekstrom E-C (2012). Women’s exposure to intimate partner violence and child malnutrition: findings from demographic and health surveys in Bangladesh. Maternal & Child Nutrition.

