# Supplementary material for: Informing the management of acute malnutrition in infants aged under 6 months (MAMI): risk factor analysis using nationally-representative demographic & health survey secondary data
Source: PeerJ. 2019 Apr 15;6:e5848. doi: 10.7717/peerj.5848 (PMC6472469; doi:10.7717/peerj.5848)
Supplement: Supplemental Information 2 [file peerj-07-5848-s002.docx]

**Characteristics of the database - Countries**

| **Country** | **Phase** | **Year** | **N** |
| --- | --- | --- | --- |
| Ghana | 5 | 2008 | 217 |
| India | 5 | 2005/06 | 3,349 |
| Kenya | 5 | 2008/09 | 494 |
| Bangladesh | 6 | 2011 | 672 |
| Burkina Faso | 6 | 2010 | 698 |
| Burundi | 6 | 2010/11 | 342 |
| Cambodia | 5 | 2010 | 301 |
| Cameroon | 6 | 2011 | 517 |
| Cote d’Ivoire | 6 | 2011/12 | 374 |
| DRC | 6 | 2013/14 | 928 |
| Egypt | 6 | 2014 | 1,210 |
| Ethiopia | 6 | 2011 | 997 |
| Malawi | 5 | 2010 | 350 |
| Mali | 6 | 2012/13 | 317 |
| Mozambique | 6 | 2011 | 936 |
| Nepal | 6 | 2011 | 215 |
| Niger | 6 | 2012 | 526 |
| Nigeria | 6 | 2013 | 2,457 |
| Pakistan | 6 | 2012/13 | 283 |
| Zambia | 6 | 2013/14 | 1,030 |
