# Supplementary material for: Informing the management of acute malnutrition in infants aged under 6 months (MAMI): risk factor analysis using nationally-representative demographic & health survey secondary data
Source: PeerJ. 2019 Apr 15;6:e5848. doi: 10.7717/peerj.5848 (PMC6472469; doi:10.7717/peerj.5848)
Supplement: Supplemental Information 3 [file peerj-07-5848-s003.docx]

Characteristics of the database

| **Sex** | **N** | **%** |
| --- | --- | --- |
| Male | 8,206 | 50.61 |
| Female | 8,007 | 49.39 |
| **Age** | **N** | **%** |
| 0 month | 1,083 | 6.68 |
| 1 month | 2,706 | 16.69 |
| 2 months | 3,043 | 18.77 |
| 3 months | 3,074 | 18.96 |
| 4 months | 3,175 | 19.58 |
| 5 months | 3,132 | 19.32 |
| **Residence** | **N** | **%** |
| Urban | 4,861 | 29.98 |
| Rural | 11,352 | 70.02 |
| Total | 16,213 | 100 |
