# Supplementary material for: Informing the management of acute malnutrition in infants aged under 6 months (MAMI): risk factor analysis using nationally-representative demographic & health survey secondary data
Source: PeerJ. 2019 Apr 15;6:e5848. doi: 10.7717/peerj.5848 (PMC6472469; doi:10.7717/peerj.5848)
Supplement: Supplemental Information 4 [file peerj-07-5848-s004.docx]

Countries not included and reason for exclusion

| Country | WHO Region | Reason for exclusion |
| --- | --- | --- |
| Afghanistan | Eastern Mediterranean | Different variable codes |
| Angola | African | Not available |
| Guatemala | Americas | Last DHS 1998 |
| Indonesia  Iraq | South-East Asian  Eastern Mediterranean | Not available  Not available |
| Madagascar | African | Weight was discarded |
| Myanmar | South-East Asian | Not available |
| Peru | Americas | Not available |
| Philippines | Western Pacific | No anthropometry |
| South Africa | African | Last DHS was in 1998 |
| Sudan | Eastern Mediterranean | Last DHS 1990 |
| Tanzania | African | No anthropometry |
| Turkey | European | Not available |
| Uganda | African | Not available |
| Vietnam | Western Pacific | Not available |
| Yemen | Eastern Mediterranean | Not available |
