# Supplementary material for: Informing the management of acute malnutrition in infants aged under 6 months (MAMI): risk factor analysis using nationally-representative demographic & health survey secondary data
Source: PeerJ. 2019 Apr 15;6:e5848. doi: 10.7717/peerj.5848 (PMC6472469; doi:10.7717/peerj.5848)
Supplement: Supplemental Information 5 [file peerj-07-5848-s005.docx]

**Household characteristics and their association with infant u6m wasting – subdivided into severe and moderate wasting**

| **Household characteristic** | | **OR** | **95% CI** | | **p-value** |
| --- | --- | --- | --- | --- | --- |
| **Residence (N=16213)** | |  |  |  |  |
| Wasting | Rural *(vs urban reference)* | 1.09 | 0.95 | 1.26 | 0.21 |
| Severe |  | 0.97 | 0.81 | 1.17 | 0.74 |
| Moderate |  | 1.18 | 0.98 | 1.43 | 0.08 |
| **Water source (N=14982)** | |  |  |  |  |
| Wasting | Non-improved *(vs. improved ref).* | 0.86 | 0.75 | 1.00 | 0.05 |
| Severe |  | 0.92 | 0.75 | 1.13 | 0.44 |
| Moderate |  | 0.84 | 0.70 | 1.01 | 0.07 |
| **Time to fetch water (N=14878)** | |  |  |  |  |
| Wasting | Source on premises | 1 | - | - | - |
|  | Source at <= 30min | 1.04 | 0.90 | 1.19 | 0.64 |
|  | Source at > 30min | 0.83 | 0.67 | 1.01 | 0.07 |
| Severe | Source on premises | 1 | - | - | - |
|  | Source at <= 30min | 0.95 | 0.78 | 1.15 | 0.58 |
|  | Source at > 30min | 0.90 | 0.67 | 1.19 | 0.45 |
| Moderate | Source on premise | 1 | - | - | - |
|  | Source at <= 30min | 1.10 | 0.92 | 1.33 | 0.29 |
|  | Source at > 30min | 0.80 | 0.62 | 1.04 | 0.09 |
| **Toilet type (N=14961)** | |  |  |  |  |
| Wasting | Improved | 1 | - | - | - |
|  | Non-improved | 0.68 | 0.57 | 0.81 | <0.001** |
|  | No toilet | 1.55 | 1.34 | 1.78 | <0.001** |
| Severe | Improved | 1 | - | - | - |
|  | Non-improved | 0.70 | 0.54 | 0.91 | 0.01* |
|  | No toilet | 1.23 | 1.01 | 1.50 | 0.04* |
| Moderate | Improved | 1 | - | - | - |
|  | Non-improved | 0.71 | 0.56 | 0.89 | <0.01** |
|  | No toilet | 1.68 | 1.40 | 2.01 | <0.001** |
| **Wealth status (N=16213)** | |  |  |  |  |
| Wasting | Middle | 1 | - | - | - |
|  | Poorest | 1.22 | 1.01 | 1.48 | 0.04* |
|  | Poorer | 1.05 | 0.86 | 1.28 | 0.62 |
|  | Richer | 0.95 | 0.78 | 1.16 | 0.63 |
|  | Richest | 0.78 | 0.63 | 0.95 | 0.02* |
| Severe | Middle | 1 | - | - | - |
|  | Poorest | 0.97 | 0.75 | 1.26 | 0.824 |
|  | Poorer | 0.92 | 0.70 | 1.22 | 0.555 |
|  | Richer | 0.90 | 0.68 | 1.19 | 0.453 |
|  | Richest | 0.67 | 0.50 | 0.89 | 0.006 |
| Moderate | Middle | 1 | - | - | - |
|  | Poorest | 1.41 | 1.11 | 1.80 | <0.01* |
|  | Poorer | 1.17 | 0.90 | 1.52 | 0.24 |
|  | Richer | 1.02 | 0.79 | 1.31 | 0.90 |
|  | Richest | 0.93 | 0.72 | 1.22 | 0.61 |
| **Own Livestock (16211)** | |  |  |  |  |
| Wasting | Has animals *(vs none ref.)* | 0.99 | 0.87 | 1.14 | 0.93 |
| Severe |  | 0.91 | 0.76 | 1.09 | 0.31 |
| Moderate |  | 1.07 | 0.90 | 1.28 | 0.44 |

*p<0.05, **p<0.01
