# Supplementary material for: Informing the management of acute malnutrition in infants aged under 6 months (MAMI): risk factor analysis using nationally-representative demographic & health survey secondary data
Source: PeerJ. 2019 Apr 15;6:e5848. doi: 10.7717/peerj.5848 (PMC6472469; doi:10.7717/peerj.5848)
Supplement: Supplemental Information 6 — *P<0.01, **P<0.001. †Adjusted for infant age group, sex and socio-economic status. [file peerj-07-5848-s006.docx]

**Maternal characteristics and their association with infant u6m wasting – subdivided into severe and moderate wasting**

|  | | **Unadjusted** | | | | **Adjusted** | | | |
| --- | --- | --- | --- | --- | --- | --- | --- | --- | --- |
|  | | **OR** | **95% CI** | | **p-value** | **OR** | **95% CI** | | **p-value** |
| **Age (N=16213)** | |  |  |  |  |  |  |  |  |
| Wasting | <20 years | 1 | - | - | - | 1 | - | - | - |
|  | >=20 & <35 | 0.99 | 0.81 | 1.20 | 0.89 | 1.00 | 0.82 | 1.22 | 0.99 |
|  | >=35 | 0.98 | 0.76 | 1.26 | 0.88 | 1.02 | 0.78 | 1.32 | 0.91 |
| Severe | <20 years | 1 | - | - | - | 1 | - | - | - |
|  | >=20 & <35 | 1.00 | 0.76 | 1.30 | 0.97 | 1.02 | 0.78 | 1.33 | 0.91 |
|  | >=35 | 0.95 | 0.68 | 1.32 | 0.75 | 0.98 | 0.70 | 1.37 | 0.90 |
| Moderate | <20 years | 1 | - | - | - | 1 | - | - | - |
|  | >=20 & <35 | 0.98 | 0.77 | 1.25 | 0.89 | 0.99 | 0.77 | 1.26 | 0.93 |
|  | >=35 | 1.01 | 0.73 | 1.40 | 0.94 | 1.04 | 0.75 | 1.46 | 0.81 |
| **BMI (N=16213)** |  |  |  |  |  |  |  |  |  |
| Wasting | >=18.5 & <25.0 (normal) | 1 | - | - | - | 1 | - | - | - |
|  | <18.5 (low) | 1.61 | 1.37 | 1.89 | <0.001** | 1.53 | 1.29 | 1.80 | <0.001** |
|  | >=25.0 & <30.0 (high) | 0.78 | 0.65 | 0.95 | 0.01* | 0.84 | 0.69 | 1.03 | 0.09 |
|  | >=30.0 (v.high) | 0.48 | 0.36 | 0.63 | <0.001** | 0.49 | 0.36 | 0.65 | <0.001** |
| Severe | >=18.5 & <25.0 (normal) | 1 | - | - | - | 1 | - | - | - |
|  | <18.5 (low) | 1.69 | 1.36 | 2.11 | <0.001** | 1.66 | 1.32 | 2.07 | <0.001** |
|  | >=25.0 & <30.0 (high) | 0.88 | 0.68 | 1.15 | 0.36 | 0.94 | 0.71 | 1.24 | 0.66 |
|  | >=30.0 (v.high) | 0.60 | 0.43 | 0.85 | <0.01** | 0.61 | 0.43 | 0.87 | <0.01** |
| Moderate | >=18.5 & <25.0 (normal) | 1 | - | - | - | 1 | - | - | - |
|  | <18.5 (low) | 1.34 | 1.10 | 1.63 | <0.01** | 1.26 | 1.03 | 1.54 | 0.03* |
|  | >=25.0 & <30.0 (high) | 0.75 | 0.58 | 0.96 | 0.02* | 0.80 | 0.62 | 1.04 | 0.093 |
|  | >=30.0 (v.high) | 0.44 | 0.29 | 0.67 | <0.001** | 0.45 | 0.30 | 0.69 | <0.001** |
| **Height (N=16213)** | |  |  |  |  |  |  |  |  |
| Wasting | <145cm(stunted)  *(vs. >=145cm ref.)* | 1.36 | 1.07 | 1.73 | 0.01* | 1.26 | 1.00 | 1.61 | 0.05 |
| Severe |  | 1.21 | 0.88 | 1.65 | 0.25 | 1.16 | 0.84 | 1.60 | 0.37 |
| Moderate |  | 1.39 | 1.01 | 1.90 | 0.04* | 1.27 | 0.93 | 1.73 | 0.14 |
| **Education (N=16211)** | |  |  |  |  |  |  |  |  |
| Wasting | No education | 1 | - | - | - | 1  (ref.) | - | - | - |
|  | Primary | 0.64 | 0.54 | 0.76 | <0.001** | 0.71 | 0.60 | 0.85 | <0.001** |
|  | Secondary | 0.86 | 0.74 | 1.00 | 0.05 | 0.97 | 0.83 | 1.15 | 0.76 |
|  | Higher | 0.64 | 0.49 | 0.84 | <0.01* | 0.80 | 0.58 | 1.10 | 0.15 |
| Severe | No education | 1 | - | - | - | 1 | - | - | - |
|  | Primary | 0.77 | 0.61 | 0.97 | 0.03* | 0.81 | 0.64 | 1.03 | 0.09 |
|  | Secondary | 0.92 | 0.75 | 1.13 | 0.45 | 1.00 | 0.80 | 1.26 | 0.98 |
|  | Higher | 0.69 | 0.47 | 1.00 | 0.05 | 0.84 | 0.55 | 1.28 | 0.43 |
| Moderate | No education | 1 | - | - | - | 1 | - | - | - |
|  | Primary | 0.61 | 0.49 | 0.76 | <0.001** | 0.69 | 0.54 | 0.86 | 0.001 |
|  | Secondary | 0.85 | 0.70 | 1.03 | 0.09 | 0.96 | 0.78 | 1.12 | 0.71 |
|  | Higher | 0.67 | 0.47 | 0.94 | 0.02* | 0.80 | 0.54 | 1.18 | 0.26 |
| **Working (N=16208)** | |  |  |  |  |  |  |  |  |
| Wasting | Working *(vs. not working ref.)* | 0.82 | 0.72 | 0.94 | <0.01** | 0.84 | 0.74 | 0.96 | 0.01 |
| Severe |  | 0.90 | 0.75 | 1.07 | 0.23 | 0.93 | 0.77 | 1.10 | 0.41 |
| Moderate |  | 0.80 | 0.68 | 0.95 | <0.01* | 0.81 | 0.69 | 0.96 | 0.02 |
| **Maternal Relationships** | |  |  |  |  |  |  |  |  |
| **In Union (N=16213)** |  |  |  |  |  |  |  |  |  |
| Wasting | Yes *(vs. not in union ref.)* | 2.09 | 1.67 | 2.62 | <0.001** | 1.89 | 1.51 | 2.37 | <0.001** |
| Severe |  | 1.73 | 1.24 | 2.43 | <0.01* | 1.66 | 1.18 | 2.34 | <0.01* |
| Moderate |  | 2.14 | 1.62 | 2.84 | <0.001** | 1.89 | 1.43 | 2.49 | <0.001** |
| **Who decides about health issues (N=15252)** | | |  |  |  |  |  |  |  |
| Wasting | Respondent | 1 | - | - | - | 1 | - | - | - |
|  | Respondent+ husband | 1.09 | 0.88 | 1.34 | 0.43 | 1.13 | 0.92 | 1.39 | 0.25 |
|  | Husband | 1.21 | 0.99 | 1.48 | 0.06 | 1.21 | 0.99 | 1.48 | 0.06 |
|  | Other | 1.49 | 1.11 | 2.00 | <0.01** | 1.42 | 1.05 | 1.92 | 0.02* |
| Severe | Respondent | 1 | - | - | - | 1 | - | - | - |
|  | Respondent+ husband | 0.97 | 0.72 | 1.30 | 0.84 | 0.98 | 0.73 | 1.32 | 0.90 |
|  | Husband | 1.09 | 0.82 | 1.44 | 0.57 | 1.07 | 0.81 | 1.42 | 0.63 |
|  | Other | 1.21 | 0.81 | 1.82 | 0.35 | 1.16 | 0.77 | 1.75 | 0.49 |
| Moderate | Respondent | 1 | - | - | - | 1 | - | - | - |
|  | Respondent+ husband | 1.18 | 0.91 | 1.52 | 0.22 | 1.24 | 0.96 | 1.60 | 0.11 |
|  | Husband | 1.27 | 0.99 | 1.64 | 0.06 | 1.28 | 0.99 | 1.65 | 0.06 |
|  | Other | 1.60 | 1.11 | 2.29 | 0.01* | 1.54 | 1.07 | 2.21 | 0.02* |
| **Emotional violence (N=8382)** | |  |  |  |  |  |  |  |  |
| Wasting | Yes *(vs. no violence ref.)* | 1.06 | 0.84 | 1.34 | 0.63 | 1.09 | 0.86 | 1.39 | 0.49 |
| Severe |  | 1.08 | 0.81 | 1.45 | 0.60 | 1.09 | 0.81 | 1.46 | 0.57 |
| Moderate |  | 1.02 | 0.78 | 1.35 | 0.87 | 1.06 | 0.80 | 1.41 | 0.69 |
| **Physical Violence (N=8381)** | |  |  |  |  |  |  |  |  |
| Wasting | Yes *(vs. no violence ref.)* | 1.34 | 1.12 | 1.62 | <0.01** | 1.30 | 1.08 | 1.57 | <0.01** |
| Severe |  | 1.47 | 1.16 | 1.86 | <0.01** | 1.45 | 1.14 | 1.84 | <0.01** |
| Moderate |  | 1.14 | 0.90 | 1.44 | 0.27 | 1.09 | 0.86 | 1.39 | 0.46 |
| **Severe Physical Violence (N=8379)** | |  |  |  |  |  |  |  |  |
| Wasting | Yes *(vs. no violence ref.)* | 1.07 | 0.80 | 1.43 | 0.63 | 1.05 | 0.78 | 1.41 | 0.76 |
| Severe |  | 0.94 | 0.63 | 1.41 | 0.78 | 0.93 | 0.61 | 1.40 | 0.72 |
| Moderate |  | 1.16 | 0.82 | 1.64 | 0.41 | 1.14 | 0.79 | 1.63 | 0.49 |
| **Sexual Violence (N=8379)** | |  |  |  |  |  |  |  |  |
| Wasting | Yes *(vs. no violence ref.)* | 1.32 | 1.00 | 1.73 | 0.05 | 1.28 | 0.96 | 1.69 | 0.09 |
| Severe |  | 1.63 | 1.16 | 2.27 | <0.01** | 1.62 | 1.15 | 2.30 | <0.01** |
| Moderate |  | 0.98 | 0.69 | 1.40 | 0.93 | 0.94 | 0.65 | 1.34 | 0.72 |
| **Any Violence (N=8623)** | |  |  |  |  |  |  |  |  |
| Wasting | Yes *(vs. no violence ref.)* | 1.28 | 1.08 | 1.52 | <0.01** | 1.26 | 1.06 | 1.50 | 0.01* |
| Severe |  | 1.39 | 1.11 | 1.74 | <0.01** | 1.38 | 1.10 | 1.73 | <0.01** |
| Moderate |  | 1.11 | 0.90 | 1.37 | 0.34 | 1.08 | 0.88 | 1.34 | 0.46 |

*p<0.05, **p<0.01
