# Supplementary material for: Informing the management of acute malnutrition in infants aged under 6 months (MAMI): risk factor analysis using nationally-representative demographic & health survey secondary data
Source: PeerJ. 2019 Apr 15;6:e5848. doi: 10.7717/peerj.5848 (PMC6472469; doi:10.7717/peerj.5848)
Supplement: Supplemental Information 7 — *P<0.01, **P<0.001. ‡This is size as reported by the carer rather than measured size. †Adjusted for infant age group, sex and socio-economic status. [file peerj-07-5848-s007.docx]

**Infant characteristics and their association with infant u6m wasting – subdivided into severe and moderate wasting**

|  | | **Unadjusted** | | | | **Adjusted** | | | |
| --- | --- | --- | --- | --- | --- | --- | --- | --- | --- |
|  | | **OR** | **95% CI** | | **p-value** | **OR** | **95% CI** | | **p-value** |
| **GENERAL CHARACTERISTICS** | |  |  |  |  |  |  |  |  |
| **Age group (N=16213)** | |  |  |  |  |  |  |  |  |
| Wasting | 0 to 2 months | 1 *(ref)* | - | - | - | 1*(ref)* | - | - | - |
|  | 3 to 5 months | 0.95 | 0.83 | 1.08 | 0.45 | 0.91 | 0.69 | 1.19 | 0.48 |
| Severe | 0 to 2 months | 1 *(ref)* |  |  |  |  |  |  |  |
|  | 3 to 5 months | 0.91 | 0.77 | 1.09 | 0.32 | 0.85 | 0.59 | 1.23 | 0.40 |
| Moderate | 0 to 2 months | 1 *(ref)* |  |  |  |  |  |  |  |
|  | 3 to 5 months | 0.99 | 0.83 | 1.18 | 0.94 | 0.98 | 0.70 | 1.38 | 0.90 |
| **Sex (N=16213)** | |  |  |  |  |  |  |  |  |
| Wasting | Female *(vs male ref)* | 1.01 | 0.89 | 1.16 | 0.83 | 1.00 | 0.88 | 1.14 | 1.00 |
| Severe | *“ “* | 0.88 | 0.74 | 1.05 | 0.16 | 0.87 | 0.73 | 1.04 | 0.13 |
| Moderate | “ “ | 1.14 | 0.96 | 1.34 | 0.13 | 1.12 | 0.95 | 1.32 | 0.17 |
| **Birth Spacing (N=12373)** | |  |  |  |  |  |  |  |  |
| Wasting | >24months *(vs <=24m ref.)* | 1.20 | 1.00 | 1.44 | 0.05 | 1.18 | 0.98 | 1.41 | 0.08 |
| Severe | “ “ | 1.24 | 0.97 | 1.59 | 0.09 | 1.23 | 0.96 | 1.57 | 0.11 |
| Moderate | “ “ | 1.11 | 0.88 | 1.40 | 0.38 | 1.09 | 0.86 | 1.37 | 0.47 |
| **Birth Order (N=16213)** | |  |  |  |  |  |  |  |  |
| Wasting | 1 (first-born) | 1 *(ref)* | - | - | - | 1 | - | - | - |
|  | 2 (second-born) | 1.04 | 0.87 | 1.25 | 0.66 | 1.02 | 0.85 | 1.23 | 0.80 |
|  | 3 (third-born) | 1.00 | 0.82 | 1.23 | 0.97 | 0.98 | 0.80 | 1.21 | 0.87 |
|  | 4 (fourth-born) | 1.06 | 0.90 | 1.27 | 0.48 | 1.00 | 0.83 | 1.22 | 0.97 |
| Severe | 1 | 1 *(ref)* | - | - | - | 1 | - | - | - |
|  | 2 | 1.17 | 0.91 | 1.50 | 0.22 | 1.15 | 0.90 | 1.48 | 0.26 |
|  | 3 | 1.07 | 0.81 | 1.41 | 0.63 | 1.05 | 0.79 | 1.39 | 0.75 |
|  | 4 | 1.06 | 0.84 | 1.33 | 0.63 | 1.00 | 0.78 | 1.29 | 0.99 |
| Moderate | 1 | 1 *(ref)* | - | - | - | 1 | - | - | - |
|  | 2 | 0.94 | 0.74 | 1.19 | 0.58 | 0.92 | 0.72 | 1.17 | 0.50 |
|  | 3 | 0.95 | 0.73 | 1.24 | 0.71 | 0.94 | 0.72 | 1.22 | 0.63 |
|  | 4 | 1.05 | 0.85 | 1.31 | 0.63 | 1.01 | 0.79 | 1.28 | 0.96 |
| **Previous child death (N=16213)** | |  |  |  |  |  |  |  |  |
| Wasting | None | 1 *(ref)* | - | - | - | 1 | - | - | - |
|  | One | 0.99 | 0.82 | 1.19 | 0.90 | 0.95 | 0.78 | 1.15 | 0.58 |
|  | Two or more | 1.08 | 0.85 | 1.36 | 0.53 | 1.00 | 0.79 | 1.28 | 0.97 |
| Severe | None | 1 *(ref)* | - | - | - | 1 | - | - | - |
|  | One | 0.90 | 0.68 | 1.18 | 0.43 | 0.87 | 0.66 | 1.15 | 0.34 |
|  | Two or more | 0.98 | 0.72 | 1.32 | 0.87 | 0.92 | 0.67 | 1.26 | 0.62 |
| Moderate | None | 1 *(ref)* | - | - | - | 1 | - | - | - |
|  | One | 1.07 | 0.84 | 1.36 | 0.60 | 1.03 | 0.80 | 1.31 | 0.84 |
|  | Two or more | 1.14 | 0.85 | 1.53 | 0.37 | 1.07 | 0.79 | 1.46 | 0.64 |
| **ANTENATAL AND BIRTH HISTORY** | |  |  |  |  |  |  |  |  |
| **Appropriate ANC 4+ visits by skilled provider (N=15908)** | |  |  |  |  |  |  |  |  |
| Wasting | *(vs not ref.)* | 0.72 | 0.64 | 0.82 | <0.001** | 0.77 | 0.67 | 0.89 | <0.001** |
| Severe |  | 0.74 | 0.62 | 0.89 | <0.01** | 0.77 | 0.63 | 0.94 | 0.01 |
| Moderate |  | 0.76 | 0.65 | 0.89 | <0.01** | 0.82 | 0.69 | 0.98 | 0.03* |
| **Born at home (N=16099)** | |  |  |  |  |  |  |  |  |
| Wasting | *(vs facility ref.)* | 1.41 | 1.23 | 1.60 | <0.001** | 1.30 | 1.12 | 1.51 | <0.01** |
| Severe |  | 1.31 | 1.10 | 1.57 | <0.01** | 1.26 | 1.03 | 1.54 | 0.03* |
| Moderate |  | 1.37 | 1.16 | 1.62 | <0.001** | 1.26 | 1.03 | 1.53 | 0.02* |
| **Born by C-section (N=16181)** | |  |  |  |  |  |  |  |  |
| Wasting | *(vs normal birth ref.)* | 0.71 | 0.57 | 0.88 | <0.01** | 0.73 | 0.58 | 0.92 | <0.01** |
| Severe |  | 0.76 | 0.57 | 1.03 | 0.08 | 0.79 | 0.57 | 1.08 | 0.13 |
| Moderate |  | 0.72 | 0.53 | 0.96 | 0.03* | 0.74 | 0.55 | 1.00 | 0.05 |
| **Size at birth (N=16035)** | |  |  |  |  |  |  |  |  |
| Wasting | Average | 1 *(ref)* | - | - | - | 1 | - | - | - |
|  | Largest | 0.83 | 0.62 | 1.09 | 0.18 | 0.89 | 0.67 | 1.18 | 0.41 |
|  | Larger than average | 0.92 | 0.76 | 1.10 | 0.33 | 0.96 | 0.80 | 1.15 | 0.64 |
|  | Smaller than average | 1.35 | 1.13 | 1.61 | <0.01** | 1.32 | 1.10 | 1.58 | <0.01** |
|  | Very Small | 1.23 | 0.98 | 1.56 | 0.08 | 1.19 | 0.94 | 1.50 | 0.15 |
| Severe | Average | 1 *(ref)* | - | - | - | 1 | - | - | - |
|  | Largest | 1.12 | 0.76 | 1.66 | 0.57 | 1.18 | 0.79 | 1.77 | 0.41 |
|  | Larger than av. | 0.89 | 0.70 | 1.13 | 0.33 | 0.92 | 0.72 | 1.17 | 0.48 |
|  | Smaller than av. | 1.11 | 0.86 | 1.43 | 0.42 | 1.09 | 0.85 | 1.41 | 0.49 |
|  | Very Small | 1.36 | 1.00 | 1.86 | 0.05 | 1.35 | 0.98 | 1.85 | 0.06 |
| Moderate | Average | 1 *(ref)* | - | - | - | 1 | - | - | - |
|  | Largest | 0.63 | 0.43 | 0.93 | 0.02* | 0.68 | 0.46 | 1.00 | 0.05 |
|  | Larger than av. | 0.96 | 0.77 | 1.19 | 0.69 | 1.00 | 0.80 | 1.25 | 0.98 |
|  | Smaller than av. | 1.45 | 1.15 | 1.81 | <0.01** | 1.40 | 1.12 | 1.76 | <0.01** |
|  | Very Small | 1.07 | 0.78 | 1.45 | 0.69 | 1.01 | 0.74 | 1.38 | 0.96 |
| **Post Natal Care (N=11258)** *Does not include phase 5 countries: Ghana, India, Kenya, Cambodia, Malawi* | |  |  |  |  |  |  |  |  |
| Wasting | *Yes (vs. no ref.)* | 0.84 | 0.73 | 0.97 | 0.02* | 0.90 | 0.77 | 1.05 | 0.17 |
| Severe |  | 0.81 | 0.65 | 0.99 | 0.04* | 0.85 | 0.68 | 1.05 | 0.14 |
| Moderate |  | 0.90 | 0.74 | 1.08 | 0.25 | 0.96 | 0.78 | 1.18 | 0.69 |
| **BREASTFEEDING RELATED** | |  |  |  |  |  |  |  |  |
| **Started BF (N=15786)** | |  |  |  |  |  |  |  |  |
| Wasting | Within 1 hr | 1 *(ref)* | - | - | - | 1 | - | - | - |
|  | Within 1 day | 1.36 | 1.17 | 1.57 | <0.001** | 1.31 | 1.13 | 1.51 | <0.001** |
|  | >1 day | 1.85 | 1.55 | 2.21 | <0.001** | 1.65 | 1.38 | 1.98 | <0.001** |
| Severe | Within 1 hr | 1 *(ref)* | - | - | - | 1 | - | - | - |
|  | Within 1 day | 1.42 | 1.16 | 1.74 | <0.01** | 1.38 | 1.13 | 1.70 | <0.01** |
|  | >1 day | 1.64 | 1.29 | 2.09 | <0.001** | 1.54 | 1.21 | 1.98 | <0.01** |
| Moderate | Within 1 hr | 1 *(ref)* | - | - | - | 1 | - | - | - |
|  | Within 1 day | 1.23 | 1.02 | 1.48 | 0.03 | 1.18 | 0.97 | 1.42 | 0.093 |
|  | >1 day | 1.76 | 1.40 | 2.21 | <0.001** | 1.54 | 1.23 | 1.93 | <0.001** |
| **Fed anything before BF (N=16213),** | |  |  |  |  |  |  |  |  |
| Wasting | *Yes (vs no ref.)* | 1.46 | 1.29 | 1.66 | <0.001** | 1.34 | 1.18 | 1.53 | <0.001** |
| Severe |  | 1.57 | 1.32 | 1.88 | <0.001** | 1.52 | 1.27 | 1.82 | <0.001** |
| Moderate |  | 1.26 | 1.07 | 1.48 | <0.01** | 1.13 | 0.96 | 1.34 | 0.14 |
| **Ever BF (N=16204)** | |  |  |  |  |  |  |  |  |
| Wasting | *Yes (vs no ref.)* | 0.38 | 0.21 | 0.70 | <0.01** | 0.36 | 0.20 | 0.65 | <0.01** |
| Severe |  | 0.36 | 0.16 | 0.79 | 0.01* | 0.35 | 0.16 | 0.74 | <0.01** |
| Moderate |  | 0.60 | 0.28 | 1.26 | 0.17 | 0.57 | 0.27 | 1.22 | 0.15 |
| **Currently BF (N=16213)** | |  |  |  |  |  |  |  |  |
| Wasting | *Yes (vs no ref.)* | 0.65 | 0.44 | 0.97 | 0.04* | 0.62 | 0.42 | 0.91 | 0.02* |
| Severe |  | 0.51 | 0.30 | 0.87 | 0.01* | 0.49 | 0.29 | 0.82 | <0.01** |
| Moderate |  | 0.96 | 0.58 | 1.60 | 0.89 | 0.92 | 0.56 | 1.53 | 0.75 |
| **Exclusively BF (N=16149)** | |  |  |  |  |  |  |  |  |
| Wasting | *Yes (vs no ref.)* | 0.89 | 0.78 | 1.03 | 0.11 | 0.84 | 0.73 | 0.97 | 0.02* |
| Severe |  | 0.90 | 0.75 | 1.08 | 0.26 | 0.84 | 0.69 | 1.01 | 0.06 |
| Moderate |  | 0.91 | 0.77 | 1.09 | 0.31 | 0.88 | 0.73 | 1.07 | 0.20 |
| **Predominantly BF (N=16071)** | |  |  |  |  |  |  |  |  |
| Wasting | *Yes (vs no ref.)* | 1.05 | 0.91 | 1.21 | 0.51 | 0.95 | 0.82 | 1.10 | 0.51 |
| Severe |  | 1.02 | 0.84 | 1.25 | 0.81 | 0.94 | 0.76 | 1.15 | 0.55 |
| Moderate |  | 1.06 | 0.88 | 1.27 | 0.56 | 0.98 | 0.81 | 1.18 | 0.8 |
| **Bottle fed yesterday (N=16201)** | |  |  |  |  |  |  |  |  |
| Wasting | *Yes (vs no ref.)* | 0.99 | 0.82 | 1.20 | 0.94 | 1.10 | 0.90 | 1.33 | 0.35 |
| Severe |  | 1.05 | 0.81 | 1.36 | 0.73 | 1.14 | 0.87 | 1.49 | 0.35 |
| Moderate |  | 0.95 | 0.75 | 1.20 | 0.66 | 1.03 | 0.81 | 1.31 | 0.78 |
|  |  |  |  |  |  |  |  |  |  |
| **VACCINE RELATED** | | |  |  |  |  |  |  |  |
| **Has vaccine card (N=16210)** | |  |  |  |  |  |  |  |  |
| Wasting | *Yes (vs no card ref.)* | 0.80 | 0.70 | 0.91 | <0.01** | 0.86 | 0.74 | 0.99 | 0.03* |
| Severe |  | 0.77 | 0.64 | 0.92 | <0.01** | 0.82 | 0.67 | 0.99 | 0.04* |
| Moderate |  | 0.87 | 0.73 | 1.02 | 0.09 | 0.93 | 0.77 | 1.12 | 0.42 |
| **Has BCG card (N=16196)** | |  |  |  |  |  |  |  |  |
| Wasting | *Yes (vs no card ref.)* | 0.79 | 0.69 | 0.90 | <0.001** | 0.85 | 0.74 | 0.97 | 0.02* |
| Severe |  | 0.79 | 0.66 | 0.95 | 0.01* | 0.85 | 0.69 | 1.03 | 0.10 |
| Moderate |  | 0.83 | 0.71 | 0.98 | 0.03* | 0.89 | 0.74 | 1.06 | 0.18 |
| **Timely vaccine recorded on card (DTP, polio) (N=16150)** | |  |  |  |  |  |  |  |  |
| Wasting | *Yes (vs not recorded ref.)* | 0.80 | 0.70 | 0.92 | <0.01** | 0.86 | 0.75 | 0.99 | 0.03* |
| Severe |  | 0.81 | 0.67 | 0.98 | 0.03* | 0.87 | 0.71 | 1.06 | 0.16 |
| Moderate |  | 0.84 | 0.71 | 0.99 | 0.04* | 0.89 | 0.74 | 1.06 | 0.20 |
| **RECENT ILLNESS EPISODES (IN LAST 2 WEEKS)** | | |  |  |  |  |  |  |  |
| **Fever (N=16197)** | |  |  |  |  |  |  |  |  |
| Wasting | *Yes (vs no ref.)* | 1.06 | 0.88 | 1.26 | 0.54 | 1.06 | 0.89 | 1.28 | 0.50 |
| Severe |  | 1.04 | 0.81 | 1.34 | 0.74 | 1.05 | 0.82 | 1.36 | 0.69 |
| Moderate |  | 1.05 | 0.85 | 1.31 | 0.64 | 1.06 | 0.84 | 1.32 | 0.63 |
| **Cough (N=16182)** | |  |  |  |  |  |  |  |  |
| Wasting | *Yes (vs no ref.)* | 0.96 | 0.82 | 1.13 | 0.63 | 0.97 | 0.83 | 1.15 | 0.76 |
| Severe |  | 0.96 | 0.76 | 1.21 | 0.71 | 0.97 | 0.76 | 1.23 | 0.8 |
| Moderate |  | 0.97 | 0.79 | 1.19 | 0.79 | 0.98 | 0.80 | 1.21 | 0.88 |
| **Sought treatment for cough/fever (N=3921)** | |  |  |  |  |  |  |  |  |
| Wasting | *Yes (vs no ref.)* | 1.11 | 0.86 | 1.44 | 0.41 | 1.15 | 0.88 | 1.50 | 0.32 |
| Severe |  | 1.15 | 0.79 | 1.68 | 0.47 | 1.17 | 0.80 | 1.73 | 0.42 |
| Moderate |  | 1.06 | 0.76 | 1.47 | 0.74 | 1.08 | 0.76 | 1.54 | 0.65 |
| **Diarrhoea (N=16200)** | |  |  |  |  |  |  |  |  |
| Wasting | *Yes (vs no ref.)* | 1.33 | 1.10 | 1.63 | <0.001** | 1.37 | 1.12 | 1.67 | <0.01** |
| Severe |  | 1.33 | 1.02 | 1.73 | 0.04* | 1.35 | 1.03 | 1.77 | 0.03* |
| Moderate |  | 1.24 | 0.97 | 1.58 | 0.09 | 1.27 | 0.99 | 1.62 | 0.06 |
| **Had ORS after diarrhoea (N=1668)** | |  |  |  |  |  |  |  |  |
| Wasting | *Yes (vs no ref.)* | 1.01 | 0.65 | 1.56 | 0.97 | 1.20 | 0.76 | 1.90 | 0.43 |
| Severe |  | 1.05 | 0.58 | 1.91 | 0.87 | 1.19 | 0.64 | 2.21 | 0.59 |
| Moderate |  | 0.97 | 0.55 | 1.73 | 0.92 | 1.15 | 0.63 | 2.08 | 0.65 |

*p<0.05, **p<0.01
